# High-Performance Lithium-Rich Layered Oxide Material: Effects of Preparation Methods on Microstructure and Electrochemical Properties

**DOI:** 10.3390/ma13020334

**Published:** 2020-01-11

**Authors:** Qiming Liu, Huali Zhu, Jun Liu, Xiongwei Liao, Zhuolin Tang, Cankai Zhou, Mengming Yuan, Junfei Duan, Lingjun Li, Zhaoyong Chen

**Affiliations:** 1 College of Materials Science and Engineering, Changsha University of Science and Technology, Changsha 410114, China; liuqiming@stu.csust.edu.cn (Q.L.); liujun@stu.csust.edu.cn (J.L.); avanda666@163.com (X.L.); tzl_edu29@163.com (Z.T.); zhoucankai@stu.csust.edu.cn (C.Z.); yuanmengming@stu.csust.edu.cn (M.Y.); junfei_duan@csust.edu.cn (J.D.); lingjun.li@csust.edu.cn (L.L.); 2 School of Physics and Electronic Science, Changsha University of Science and Technology, Changsha 410114, China; juliezhu2005@126.com

**Keywords:** lithium-rich layered oxide, cathode material, 0.5Li_2_MnO_3_·0.5LiMn_0.8_Ni_0.1_Co_0.1_O_2_, voltage decay, co-precipitation method, sol–gel method

## Abstract

Lithium-rich layered oxide is one of the most promising candidates for the next-generation cathode materials of high-energy-density lithium ion batteries because of its high discharge capacity. However, it has the disadvantages of uneven composition, voltage decay, and poor rate capacity, which are closely related to the preparation method. Here, 0.5Li_2_MnO_3_·0.5LiMn_0.8_Ni_0.1_Co_0.1_O_2_ was successfully prepared by sol–gel and oxalate co-precipitation methods. A systematic analysis of the materials shows that the 0.5Li_2_MnO_3_·0.5LiMn_0.8_Ni_0.1_Co_0.1_O_2_ prepared by the oxalic acid co-precipitation method had the most stable layered structure and the best electrochemical performance. The initial discharge specific capacity was 261.6 mAh·g^−1^ at 0.05 C, and the discharge specific capacity was 138 mAh·g^−1^ at 5 C. The voltage decay was only 210 mV, and the capacity retention was 94.2% after 100 cycles at 1 C. The suppression of voltage decay can be attributed to the high nickel content and uniform element distribution. In addition, tightly packed porous spheres help to reduce lithium ion diffusion energy and improve the stability of the layered structure, thereby improving cycle stability and rate capacity. This conclusion provides a reference for designing high-energy-density lithium-ion batteries.

## 1. Introduction

With the rapid development of hybrid vehicles and electric vehicles, lithium-ion batteries have been more widely used [1,2,3,4]. However, the traditional lithium-ion battery cathode materials (e.g., LiMn_2_O_4_, LiFePO_4_, LiNi_1/3_Co_1/3_Mn_1/3_O_2_, LiNi_0.5_Co_0.2_Mn_0.3_O_2_) have a discharge capacity of less than 200 mAh·g^−1^, which cannot meet the development requirements of the electric vehicle industry [5,6,7]. The current solution is to increase the nickel content or use lithium-rich layered oxides to increase the discharge capacity [8,9,10,11,12]. The lithium-rich layered oxide has become a candidate for the next generation of high-energy density lithium-ion battery cathode materials due to its high capacity and high operating voltage [13,14]. However, before commercialization, the technical challenge of voltage decay must be addressed [15,16,17,18].

Many studies have shown that ion doping [19,20,21] and surface coating [22,23,24] can suppress voltage decay of lithium-rich layered oxides. In addition, increasing the nickel content in the lithium-rich layered oxide can also significantly suppress the voltage decay, and the energy density is also improved [25,26,27]. The preparation methods of lithium-rich layered oxides include solid-state [28], co-precipitation [25], sol–gel [29], solvothermal [30], freeze drying [31], and bubble template [32]. It is controversial whether the lithium-rich layered oxide is a solid solution or a two-phase composite structure, and the structure and performance of the lithium-rich layered oxide are closely related to the synthesis [33,34,35]. In addition, the preparation method also affects the atomic spatial uniformity of chemical species [36]. The effect of lithium-rich layered oxides with low nickel content on the synthesis method has been studied intensively [37,38,39]. However, for lithium-rich layered oxides with high nickel content, such as 0.5Li_2_MnO_3_·0.5LiNi_0.8_Co_0.1_Mn_0.1_O_2_ (LL-811), the effects of the preparation method on the microstructure and electrochemical performance and the reasons for the decreased discharge capacity with increasing nickel content have not been investigated.

In this work, we compared the effects of the sol–gel method and the oxalate co-precipitation method on the microstructure, element distribution, and electrochemical performance of LL-811. Two kinds of typical chelating agents, citric acid and sucrose, were selected in the sol–gel method. LL-811 prepared by the oxalate co-precipitation method had the best comprehensive performance. After 100 cycles at 1 C, the voltage decay was 210 mV, and the capacity retention was 94.2%. The discharge specific capacity still reached 138 mAh·g^−1^ at 5 C. The significant reduction in voltage decay can be attributed to the high nickel content and uniform element distribution. In addition, tightly packed porous spheres contributed to reducing the lithium ion diffusion energy barrier and improving cycle stability and rate capacity.

## 2. Experiment

### 2.1. The Reagents and Materials

Lithium acetate dihydrate (Li(CH_3_COO)·2H_2_O, 99.0%), manganese acetate tetrahydrate (Mn(CH_3_COO)_2_·4H_2_O, 99.0%), cobalt acetate tetrahydrate (Co(CH_3_COO)_2_·4H_2_O, 99.5%), nickel acetate tetrahydrate (Ni(CH_3_COO)_2_·4H_2_O, 98.0%), ammonium oxalate monohydrate (C_2_H_8_N_2_O_4_·H_2_O, 99.5%), oxalic acid dihydrate (C_2_H_2_O_4_·2H_2_O, 99.5%), sucrose (C_12_H_22_O_11_, 99.0%), citric acid monohydrate (C_6_H_8_O_7_·H_2_O, 99.5%), sodium hydroxide (NaOH, 96.0%), nitric acid (HNO_3_, 65–68%), and ammonium hydroxide aqueous solution (NH_3_·H_2_O, 26.7%) were purchased from Sinopharm Chemical Reagent (Shanghai, China). Lithium hydroxide monohydrate (LiOH·H_2_O, 98.0%) was purchased from Xilong Scientific Co., Ltd. (Shantou, China). Nickel sulfate hexahydrate (NiSO_4_·6H_2_O, 22 wt%) was purchased from Jinchuan group Co., Ltd. (Jinchang, China). Cobalt sulfate heptahydrate (CoSO_4_·4H_2_O, 21 wt%) was purchased from Huayou Cobalt Co., Ltd. (Jiangxing, China) Manganese sulfate monohydrate (MnSO_4_·H_2_O, 31.8 wt%) was purchased from ISKY Chemicals Co., Ltd. (Changsha, China).

### 2.2. Sol–Gel Method

LL-811 was synthesized by sol–gel method, using acetic acid salts as raw materials and citric acid monohydrate as a chelating agent. Firstly, according to the stoichiometric ratio, lithium acetate dihydrate, nickel acetate tetrahydrate, cobalt acetate tetrahydrate, and manganese acetate tetrahydrate were added to deionized water to prepare a solution A of 1.5 mol/L. citric acid monohydrate was added to deionized water and configured to a concentration of 2 mol/L solution B. The molar ratio of metal ion to chelating agent was fixed to be 1:1.5. Then, the A and B solutions were slowly mixed, and the pH of the solution was adjusted to 7–8 with ammonium hydroxide. The resulting solution was evaporated at 80 °C in a constant temperature water bath to form a transparent xerogel. The gel was dried in a forced air oven at 120 °C for 12 h to obtain a precursor. Finally, the precursor was calcined at 480 °C for 5 h and then calcined at 850 °C for 12 h in air to obtain a lithium-rich layered oxide (labeled as SLC). When using the same procedure, the chelating agent was changed to sucrose, and the pH was adjusted to 5 with dilute nitric acid. The acquired sample was labeled as SLS.

### 2.3. Co-Precipitation Method

LL-811 was synthesized by oxalate co-precipitation. Firstly, according to the stoichiometric ratio, nickel sulfate hexahydrate, cobalt sulfate heptahydrate, and manganese sulfate monohydrate were added to deionized water to prepare a solution C of 1.0 mol/L. Ammonium oxalate monohydrate and oxalic acid dihydrate were added to deionized water to configure a solution D with an oxalate concentration of 0.6 mol/L. The ratios of the amounts of the transition metal salts to the substances of the complexing agent and the precipitating agent were 1:2 and 1:1.2, respectively. Secondly, the solution C and the solution D were simultaneously slowly pumped into a 5 L continuous stirred-tank reactor (CSTR). The temperature and agitation speed were maintained at 50 °C and 800 rpm, respectively. In the co-precipitation process, the pH of the solution was maintained at 6.6 by the addition of 1 mol/L sodium hydroxide solution. After the feed was completed, stirring was continued for 1 h to allow the metal ions to completely precipitate. In order to prevent oxidation of the metal cations throughout the process, the CSTR was maintained under a nitrogen atmosphere. Finally, the prepared oxalate precursor was acquired by vacuum filtration and washed with deionized water and then dried in a drying oven at 80 °C for 12 h. The prepared oxalate precursor was thoroughly mixed with 5 wt% excess of LiOH·H_2_O powder. The mixture was transferred to a furnace and calcined at 480 °C for 5 h and then calcined at 850 °C for 12 h in air to obtain a lithium-rich layered oxide (labeled as OCP).

### 2.4. Materials Characterization

The crystal structure of LL-811 was observed with an X-ray diffractometer (XRD, Bruker AXS D8 Advance, Bruker Corporation, Karlsruhe, Germany). The morphology was characterized by field emission scanning electron microscopy (SEM, TESCAN MIRA3 LMU, TESCAN, Brno, Czech Republic). The elemental compositions were characterized using energy dispersive spectroscopy (EDS, Oxford X-Max20, Oxford, UK). The analysis of the microstructure and composition of LL-811 was performed on a transmission electron microscope (TEM, Tecnai G2 F20, Hillsboro, OR, USA) equipped with energy dispersive X-rays (EDX).

The preparation of the positive electrode sheet, the assembly of the button cell, and the test procedures for electrochemical performance were consistent with the previously published articles [40]. The electrolyte is 1 M LiPF_6_, which is soluble in ethyl carbonate (EC) and dimethyl carbonate (DMC) (1:1 by volume, CAPCHEM, Shenzhen, China). The load of the active material on the positive electrode sheet was about 2.7 mg·cm^−2^. The CT-4008 battery testing system of NEWARE (Shenzhen, China) was used to test charge and discharge performance of the battery. Electrochemical impedance spectroscopy was performed with a CHI660E electrochemical workstation (Chinstruments, Shanghai, China) in the frequency range of 1 mHz to 100 kHz.

## 3. Results and Discussion

**Figure 1:**
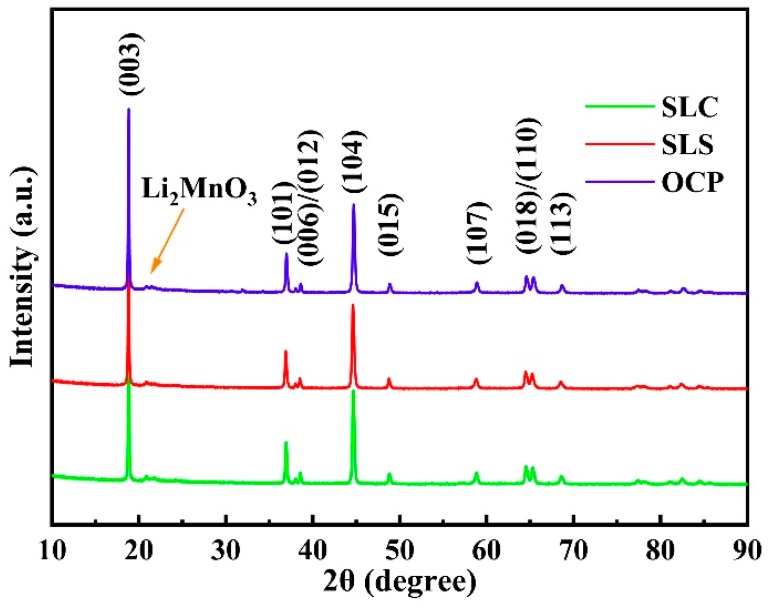
XRD patterns of LL-811 samples prepared by different methods.

The powder X-ray diffraction patterns of the lithium-rich layered oxide prepared by two different methods are shown in Figure 1. The major peaks in the XRD pattern could be indexed based on α-NaFeO_2_ layered structure with space group R$\bar{3}$m and monoclinic symmetry with space group C2/m [32]. The (006)/(012) peak and (018)/(110) peak can be clearly distinguished, indicating that the material is a typical layered structure [41]. The lattice parameters of the prepared samples were analyzed via JADE6.0, as shown in Table 1. Compared with SLS and SLC, c and c/a values increased in OCP, indicating that the crystal lattice preferentially grows along the *c* axis, thereby promoting the electrochemical reaction.

**Figure 2:**
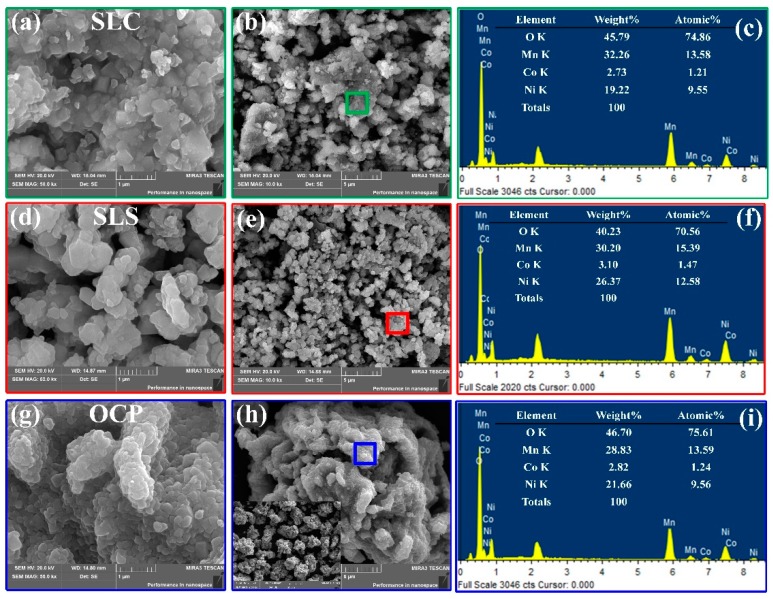
SEM images and energy dispersion spectra (EDS): (**a**–**c**) SLC; (**d**–**f**) SLS; (**g**–**i**) OCP.

The SEM micrographs and elemental compositions of samples SLC, SLS, and OCP are shown in Figure 2. As shown in Figure 2a,b,d,e, the primary particles of the samples SLC and SLS have a size of about 400–600 nm. The agglomerated secondary particles have no obvious morphology. As shown in Figure 2g, the primary particle size of the sample OCP is about 300–500 nm. As can be seen from Figure 2h, the secondary particles are spheroidal and have a particle size of about 20 μm. The embedded image in Figure 2h shows the SEM image of OCP secondary particles at low magnification. In addition, there are pores on the surface of the secondary particles closely clustered by the primary particles. Figure 2c,f,i shows the energy dispersion spectra (EDS) of SLC, SLS, and OCP, respectively. All peaks correspond to the characteristic peaks of the O, Mn, Co, and Ni elements, and the ratios of the elements are shown in the interpolation table. It can be clearly seen that there are differences in the element ratios of the three samples, especially the Ni and Mn. This may be the effect of ammonium radicals because in the reaction system, ammonium radicals complex with metal ions, which affects the chelation reaction of citric acid and metal ions and the precipitation reaction of oxalate and metal ions. In addition, the pH value may also affect the elemental composition of the product and the uniformity of the element distribution. These results indicate that a lithium-rich layered oxide positive electrode material with a high nickel content has been acquired.

**Figure 3:**
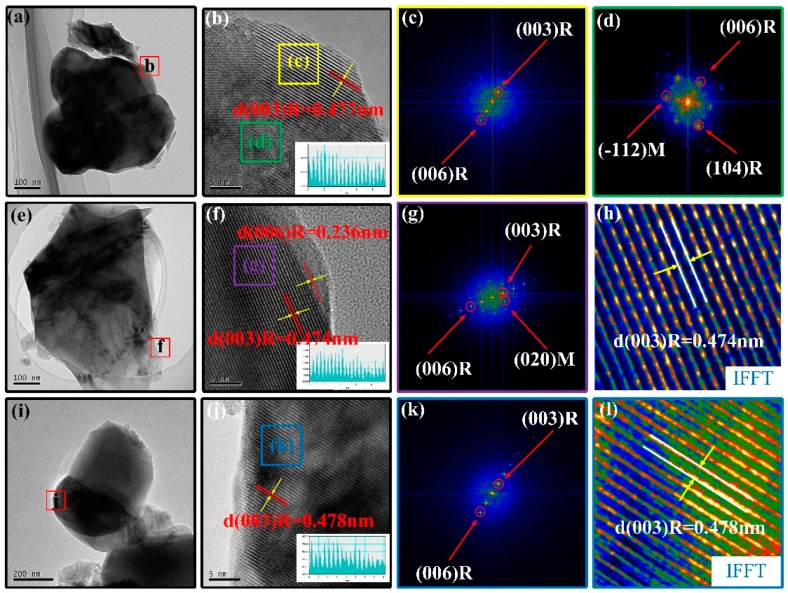
TEM and HRTEM images: (**a**,**b**) SLC; (**e**,**f**) SLS; (**i**,**j**) OCP; (**c**,**d**) fast Fourier transform (FFT) of the corresponding area in (**b**); (**g**,**h**) FFT and inverse fast Fourier transform (IFFT) of the corresponding area in (**f**); (**k**,**l**) FFT and IFFT of the corresponding area in (**j**). The indexes marked by R and M are related to the rhombohedral (R$\bar{3}$m) phase and monoclinic Li_2_MnO_3_ (C2/m) phase.

**Figure 4:**
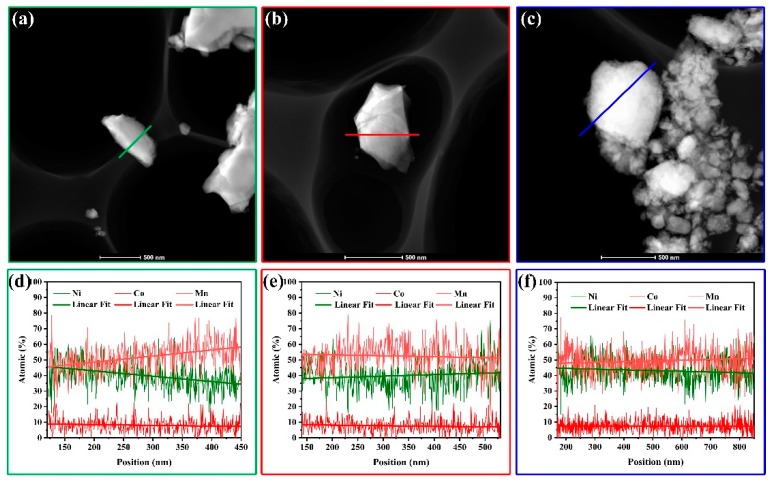
TEM images and X-ray line scan element distribution (EDX) maps: (**a**,**d**) SLC; (**b**,**e**) SLS; (**c**,**f**) OCP.

To further understand the effect of preparation methods on crystal microstructure, the microscopic morphology of the samples SLC, SLS, and OCP are shown in Figure 3. Figure 3a,e,i shows TEM images of SLC, SLS, and OCP samples, respectively, and the particle size is consistent with that of the SEM image. Figure 3b,f,j shows the HRTEM images of the corresponding area in Figure 3a,e,i. There is a clear lattice fringe in the “c” region of Figure 3b with interplanar spacing of 0.477 nm, corresponding to the (003) plane of the layered phase. These corresponding diffraction points of the (003) and (006) panel can be found in Figure 3c of its fast Fourier transform (FFT). The FFT of the “d” region in Figure 3b is shown in Figure 3d, and there are diffraction points of the α-NaFeO_2_ layered structure of the space group R$\bar{3}$m and the monoclinic system of the space group C2/m. Figure 3f shows very clear lattice fringes with a lattice spacing of 0.474 nm and 0.236 nm, corresponding to the (003) and (006) plane of the α-NaFeO_2_ layered structure, respectively. The FFT pattern and inverse fast Fourier transform (IFFT) pattern of the “g” region are shown in Figure 3g,h. The lattice spacing measured in Figure 3j is 0.478 nm, corresponding to the (003) plane in the layered structure. Figure 3k,l shows the FFT pattern and IFFT pattern of the “k” region, respectively. These isolated two sets of lattice pattern of LiMO_2_ and Li_2_MnO_3_ indicate lithium-rich layered oxides have composite structure in SLC and SLS samples, while the only one set of lattice pattern gives proof of solid solution in the OCP sample. Moreover, for OCP samples, the (003) plane has been significantly expanded compared to the other two samples, and it helps to improve the rate capacity of lithium-rich layered oxides. X-ray line scan element distribution (EDX) maps of SLC, SLS, and OCP samples are shown in Figure 4. The results show that the elements of OCP are uniformly distributed, and no nickel segregation occurs [36,42]. Evenly distributed transition metal elements help to enhance the stability of the layered structure and suppress capacity and voltage decay [21,36].

**Figure 5:**
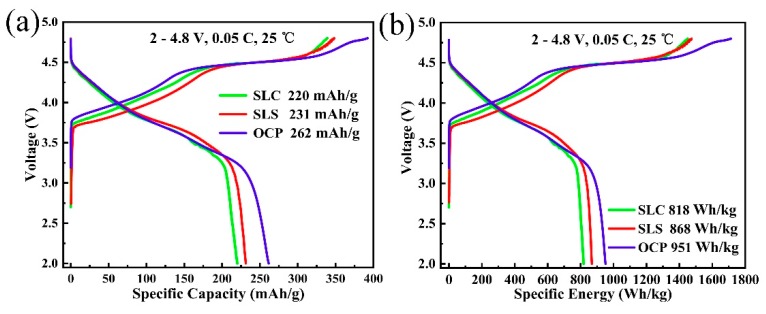
Initial charge and discharge curves of LL-811 samples prepared by different methods: (**a**) specific capacity; (**b**) specific energy.

The initial charge and discharge curves for all samples between 2.0 and 4.8 V at the current rate of 0.05 C are shown in Figure 5a. It shows similar initial charge and discharge curves, which is consistent with the characteristic curve of the lithium-rich layered oxide. The charging curve can be divided into an “S” zone below 4.5 V and an “L” zone above 4.5 V. The “S” region corresponds to the oxidation of the transition metal in the LiNi_0.8_Co_0.1_Mn_0.1_O_2_ component, and the “L” region corresponds to the Li and O removed from the crystal structure in the form of “Li_2_O” [25]. As shown in Figure 5a, the initial discharge specific capacity of LL-811 prepared by the co-precipitation method is 262 mAh·g^−1^, which is much higher than that of the SLC (220 mAh·g^−1^) and SLS (231 mAh·g^−1^) prepared by the sol–gel method. From the charging curve, the OCP sample has a longer 4.5 V platform compared to the SLC and SLS samples, which indicates that more non-electrochemically active Li_2_MnO_3_ is activated. According to the analysis of the TEM image (Figure 3), compared with the two-phase composite structure of SLC and SLS, the solid solution structure of OCP may be favorable for activating Li_2_MnO_3_. Figure 5b shows the initial charge–discharge curve of specific energy. OCP has the highest specific capacity, which is attributed to the fact that the lithium-rich layered oxide prepared by the oxalate co-precipitation method has smaller primary particles, and the secondary particles have a porous spherical morphology, which can shorten the diffusion pathway of Li^+^ ions.

**Figure 6:**
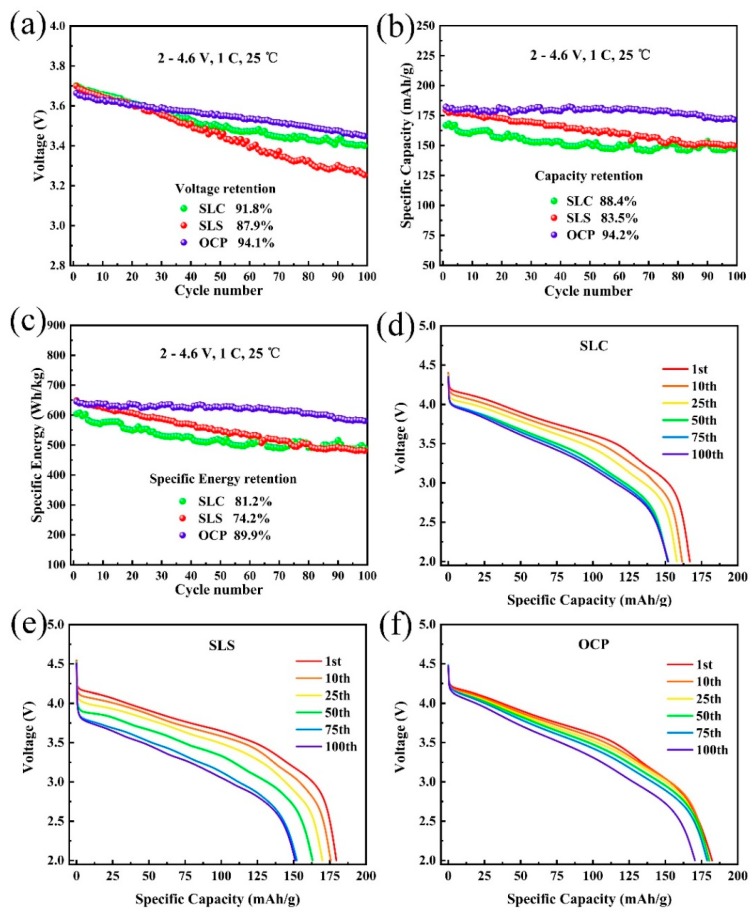
Voltage and capacity decay of LL-811 samples prepared by different methods: (**a**) voltage decay, (**b**) specific capacity decay, (**c**) specific energy decay, (**d**–**f**) discharge curves for different cycles.

Figure 6 shows the voltage and capacity decay of all samples between 2.0–4.6 V at a current rate of 1 C. As can be seen from Figure 6a, after 100 cycles, the discharge median voltage decay of OCP is 210 mV and the retention reaches 94.1%, which is higher than 91.8% of SLC and 87.9% of SLS, and the voltage decay is obviously suppressed. Figure 6b shows the specific capacity decay of the lithium-rich layered oxide cathode material. It can be seen that after 100 cycles, the specific capacity retention of OCP is 94.2%, which is significantly higher than 88.4% of SLC and 83.5% of SLS. By comparing the discharge specific energy of all samples, the results in Figure 6c are similar to those in Figure 6b. The discharge specific energy of OCP retention is 89.9%, while the SLC and SLS are only 81.2% and 74.2%, respectively. Figure 6d–f shows the discharge curves of SLC, SLS, and OCP at different cycle times. In the first 75 cycles, the specific capacities of the SLC and SLS were decayed from 166.9 mAh·g^−1^ to 152.1 mAh·g^−1^ and 179.5 mAh·g^−1^ to 152.2 mAh·g^−1^, respectively. However, the specific capacity of OCP was only decayed from 182.3 mAh·g^−1^ to 178.7 mAh·g^−1^, and the capacity decay is much smaller than SLC and SLS. For the discharge median voltage, the decay of OCP is also significantly suppressed. OCP has a lowest voltage decay and a larger specific capacity after 75 cycles, indicating that its layered structure is more stable. The voltage and capacity decay of the OCP samples was suppressed, which can be attributed to the high nickel content, uniform element distribution, and stable layered structure [25,26,36]. In addition, the secondary particle structure is tight, and the presence of pores on the surface is also the reason for the best electrochemical performance of OCP.

**Figure 7:**
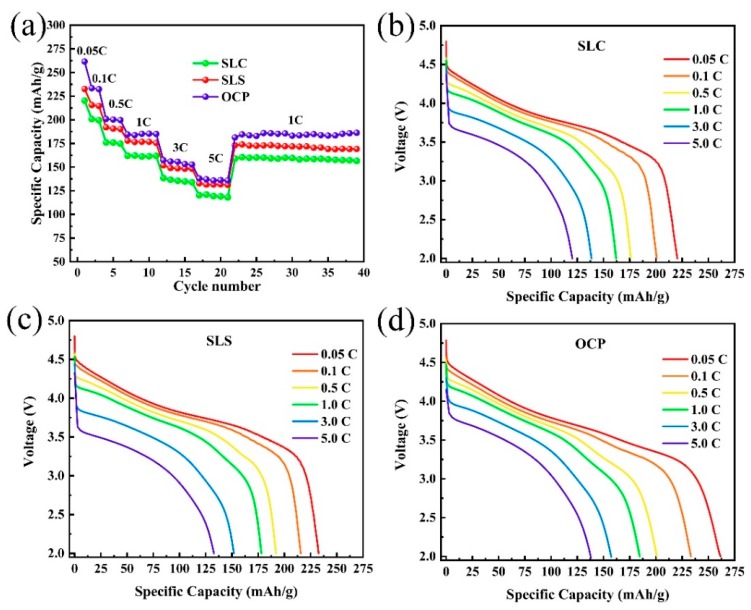
Rate performance of LL-811 samples prepared by different methods: (**a**) rate capacity, (**b**–**d**) discharge curves at different rates.

To further understand the effect of the preparation method on the rate capability, the rate capability of SLC, SLS, and OCP is shown in Figure 7. The samples prepared by oxalate co-precipitation show higher discharge capacity at various rates. The specific capacities of OCP are 261.6, 233.4, 200.9, 184.6, 157.4, and 138.0 mAh·g^−1^ at the discharge rates of 0.05, 0.1, 0.5, 1, 3, and 5 C, respectively. This indicates that increasing the spacing of the (003) plane, increasing the contact area of the electrolyte with the positive electrode material, and shortening the lithium ion diffusion path can increase the rate capacity of the lithium-rich layered oxide. In addition, the solid solution structure may also help increase the specific capacity of lithium-rich layered oxides. Figure 7b,c shows the discharge curves of SLC, SLS, and OCP at different discharge rates. As the rates increase, the discharge capacity and voltage of all samples have different degrees of decay. The results show that when the battery is discharged at a higher current density, the electrode resistance increases significantly, and the discharge energy is greatly reduced, which seriously affects the application of the lithium-rich layered oxide positive electrode material in electric vehicles. Although the OCP sample has the highest specific capacity under different current densities, its specific capacity has a greater decay at large current densities, resulting in a lower rate capacity retention than the SLS sample.

**Figure 8:**
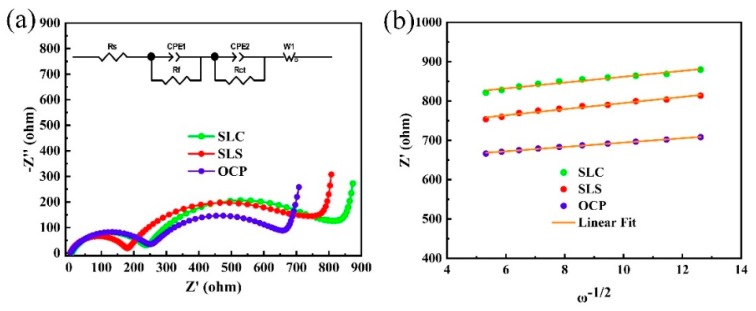
(**a**) Nyquist plots and (**b**) plots comparison of Z’ vs ω^−1/2^ for LL-811 samples after the first cycle.

In order to understand the influence of the preparation method on the interfacial electrochemical and reaction kinetics of LL-811, the electrochemical impedance spectroscopy (EIS) of LL-811 prepared by different preparation methods were investigated. The Nyquist plots of SLC, SLS, and OCP are shown in Figure 8a. The impedance spectrum was fitted using the embedded equivalent circuit in Figure 8a, and the fitting results are shown in Table 2. The results show that the SLS has the smallest Rf and OCP has the smallest charge-transfer resistance (Rct). Figure 8b shows the linear relationship between Z′ and ω^−1/2^, and the slope obtained by linear fitting represents the value of σ. The lithium ion diffusion coefficients calculated by the formula are shown in Table 2 [43]. The lithium ion diffusion coefficient of OCP is 3.67 × 10^−13^ cm^2^·s^−1^, which is higher than 2.04 × 10^−13^ cm^2^·s^−1^ of SLC and 1.86 × 10^−13^ cm^2^·s^−1^ of SLS. This proves that the lithium-rich layered oxide prepared by the oxalic acid co-precipitation method has a faster migration rate of lithium ions, and the rate performance is excellent in comparison.

## 4. Conclusions

The effects of preparation methods on the structure, morphology, and electrochemical properties of 0.5Li_2_MnO_3_·0.5LiMn_0.8_Ni_0.1_Co_0.1_O_2_ cathode materials were systematically investigated. The results show that the lithium-rich layered oxide prepared by the oxalate co-precipitation method has the best performance. After 100 cycles at 1 C, the voltage and capacity decayed were only 210 mV and 10 mAh·g^−1^, and the retention rates were 94.1% and 94.2%, respectively. The specific capacities of OCP are 261.6, 233.4, 200.9, 184.6, 157.4, and 138.0 mAh·g^−1^ at the discharge rates of 0.05, 0.1, 0.5, 1, 3, and 5 C, respectively. The significant reduction in voltage decay can be attributed to the high nickel content and uniform element distribution. In addition, tightly packed porous spheres help to reduce lithium ion diffusion energy and improve cycle stability and rate capacity. Therefore, the synthesis method plays an important role in the preparation of high-energy-density lithium-rich layered oxide cathode materials. This conclusion provides a reference for designing high-energy-density lithium-ion batteries.

## Figures and Tables

**Table 1:** Lattice parameters of LL-811 samples prepared by different methods.

Sample	Lattice Parameters			c/a
	a (Å)	c (Å)	v (Å^3^)	
SLC	2.8564	14.2203	100.49	4.978
SLS	2.8605	14.2348	100.87	4.976
OCP	2.8551	14.2376	100.37	4.980

**Table 2:** Impedance and lithium ion diffusion coefficient of LL-811 prepared by different methods.

Sample	R_f_ (Ω)	Rct (Ω)	D_Li_^+^ (cm^2^·s^−1^)
SLC	230	503	2.04 × 10^−13^
SLS	173	491	1.86 × 10^−13^
OCP	246	369	3.67 × 10^−13^
